# The Influence of Aerobic Fitness on Heart Rate Responses of Custody Assistant Recruits during Circuit Training Sessions

**DOI:** 10.3390/ijerph17218177

**Published:** 2020-11-05

**Authors:** Matthew R. Moreno, Karly A. Rodas, Ashley M. Bloodgood, J. Jay Dawes, Joseph M. Dulla, Robin M. Orr, Robert G. Lockie

**Affiliations:** 1 Department of Kinesiology, California State University, Fullerton, CA 92835, USA; moreno.matthewr@csu.fullerton.edu (M.R.M.); cesariokarly@csu.fullerton.edu (K.A.R.); abloodgood17@csu.fullerton.edu (A.M.B.); 2 School of Kinesiology, Applied Health and Recreation, Oklahoma State University, Stillwater, OK 74078, USA; jay.dawes@okstate.edu; 3 Tactical Research Unit, Bond University, Robina, QLD 4229, Australia; joseph.dulla@student.bond.edu.au (J.M.D.); rorr@bond.edu.au (R.M.O.)

**Keywords:** academy, aerobic fitness, correctional, law enforcement, police, tactical, training intensity, YMCA step test

## Abstract

This study captured heart rate (HR) responses of custody assistant (CA) recruits undertaking circuit training sessions. Data from 10 male and 12 female CA recruits were analyzed. Based on YMCA step test recovery HR, recruits were divided into higher fitness (HF; top 25%), lower fitness (LF; bottom 25%), and moderate fitness (MF; remaining recruits) groups. HR was measured during two circuit training sessions featuring calisthenics and running. HR zones were defined as: very light (<57% of age-predicted maximum heart-rate [HRmax]); light (57–63% HRmax); moderate (64–76% HRmax); vigorous (77–95% HRmax); and very vigorous (>95% HRmax). A one-way ANOVA, with Bonferroni post hoc, calculated between-group differences in time spent, and percentage of total time, in the HR zones. In session one, the LF group spent less time in the light training zone compared to the MF group, and more time in the very vigorous zone compared to the HF group (*p* = 0.027–0.047). In session two, the LF group spent more time in the moderate zone compared to both groups, and a greater percentage of time in the very vigorous zone compared to the MF group (*p* = 0.002–0.004). LF recruits generally worked harder during circuit training than their fitter counterparts, which supported recommendations for ability-based modifications.

## 1. Introduction

Law enforcement agencies provide a range of potential jobs for people from the general population. The most notable are peace or law enforcement officer positions. However, another important position within an agency is that of a civilian jailor or custody assistant (CA) [1]. People in these positions are responsible for assisting officers with maintaining order and security in custody detention, station jails, or court lockup facilities [1,2,3]. This could encompass the searching of cells, responding to alarms to assist colleagues, physical confrontations which could involve control and restraint of an inmate, or the need to pursue and corral an inmate attempting to evade capture [4,5]. Indeed, physical altercations with inmates can be extremely demanding. During an approximate 40-second defensive tactics training scenario, Lockie et al. [6] found CA recruits can achieve a heart rate (HR) greater than 90% of their age-predicted maximum heart rate. Accordingly, CAs need some level of physical fitness in order to perform their job tasks successfully, such that they can ensure the safety of themselves, their colleagues, and inmates.

Similar to other tactical professionals (police, firefighting, and the military), CA and correctional recruits will often need to complete academy training prior to employment. Specific to law enforcement, academy training is used to physically and mentally prepare recruits for the demands of the occupation [7,8,9]. A component of academy is physical training, which is in part utilized to develop the fitness characteristics important for performing job tasks. Some of the important characteristics for CAs include muscular endurance, strength, and power, and anaerobic and aerobic fitness [1,2,3,4,5,10]. It would be beneficial for training staff to enhance these qualities in their recruits before they graduate from academy. Further, numerous studies have detailed the importance of fitness for successfully graduating from a law enforcement training academy [11,12,13,14,15,16,17]. For example, law enforcement recruits who graduate from academy tend to have superior aerobic fitness as measured by the 2.4 km (1.5 mile) run [12,13] and multistage fitness test [11,14,17] compared to those recruits who did not finish academy.

Training staff encounter many challenges when conducting their physical training sessions during academy, including space and equipment limitations, and a high recruit-to-staff ratio [18]. Accordingly, staff often use training modalities that can be completed by one group, or a limited number of groups, within a restricted space without the need for traditional gym equipment. One example is circuit training [19,20,21], which involves performing a series of resistance-based exercises consecutively with minimal rest periods between each exercise [22]. This approach can be beneficial in tactical environments as a limited quantity of equipment can be adapted to specific training sites (e.g., weighted vests, sandbags, and ammunition cans can be used in place of barbells and dumbbells) [19,23]. Circuit training has the added benefit of potentially developing several fitness qualities concurrently in trained [24] and untrained [25] populations, although a primary focus is often aerobic conditioning [26]. Given the value of aerobic fitness for custody job tasks [4,5] and academy graduation [11,12,13,14,17], it would be beneficial for CA recruits to use circuit training for aerobic fitness development.

However, law enforcement academies may not always utilize optimal training practices to develop aerobic fitness in their recruits. Indeed, many training academies operate via a paramilitary, “one-size-fits-all” model [1,3,7,9,14,19,27]. The expectation from this type of training model is that all recruits complete the same exercises, with limited individual modifications for exercise type, work-to-rest ratio, volume, and intensity. The paramilitary training model has been adopted partly because the job tasks of law enforcement personnel are often the same, regardless of sex, age, and ability [8]. However, circuit training completed within a “one-sized-fits-all” model may not be the best way to develop fitness across a recruit class. Several studies have noted wide fitness variation across academy classes in law enforcement recruits [9,28], which is also reflected in CA recruit classes [3]. Another issue is that custody positions do not always feature any pre-hire fitness testing [1,2,3]. This means that CA recruits may not always start academy in the best physical condition. Cesario et al. [29] revealed that CA recruits from three academy classes group generally had an overall fitness level (measured by tests such as the one-minute push-up and sit-up test, grip strength, and the YMCA step test) that was lesser than the general population. If a circuit training session is administered with limited ability-based modifications, different exercise intensities could be experienced by individual recruits. This may not optimize fitness improvements for most CA recruits across a training academy. While many practitioners may assume this, it is essential to specifically detail this in CA recruit populations. This is important given that culture and tradition may drive training approaches in law enforcement academies [18,30], potentially more so than the best training practices. If research can detail differences in exercise intensity experienced by CA recruits across the same training session, this would help build evidence in support of ability-based circuit training.

There has been limited analysis of the training practices adopted for CA recruits, and physical training for CA recruits can often follow traditional, “one-sized-fits-all” models that have limited ability-based modifications [1,3]. This is potentially problematic, as CA recruits typically do not complete pre-hire fitness testing [1,2,3], and tend to demonstrate lesser fitness compared to the general population [29]. Given the range of fitness capabilities present in CA recruits [3], it is essential to demonstrate the HR responses of CA recruits to the imposed demands of modalities such as circuit training. This would detail whether the training practices are appropriate for individual CA recruits. Accordingly, this study detailed the HR responses of CA recruits from one academy to two circuit training sessions. Recruits were grouped as higher fitness (HF), moderate fitness (MF), or lower fitness (LF) as defined by recovery HR from the YMCA step test to determine how exercise intensity varied within a class. It was hypothesized that the HF recruits would be working at a lower intensity during the sessions, while LF recruits would be working at a higher intensity.

## 2. Materials and Methods

### 2.1. Subjects

Data from 22 CA recruits (age = 27.59 ± 6.62 years; height = 1.68 ± 0.06 m; body mass = 67.68 ± 11.09 kg), encompassing 10 men (age = 28.20 ± 5.49 years; height = 1.72 ± 0.06 m; body mass = 73.59 ± 8.75 kg) and 12 women (age = 27.08 ± 7.63 years; height = 1.65 ± 0.05 m; body mass = 62.76 ± 10.68 kg), from a one academy class were analyzed. This was a convenience sample provided by the law enforcement agency. As a result, the researchers had no control of the final sample size used in the study. For recruits to be included, the had to be part of the CA academy class and have full data sets from both circuit training sessions. Recruits were excluded if they had incomplete data sets from either of the circuit training sessions that were analyzed. The characteristics of the subjects in this study, and the between-sex ratio, was similar to that from previous CA research [1,3]. Although the sample size was relatively small, it was similar to actual CA academy class numbers shown in the literature [3]. Based on the archival nature of this study, the institutional ethics committee approved the use of pre-existing data (HSR-17-18-370). The study also conformed to the recommendations from the Declaration of Helsinki [31].

### 2.2. Procedures

The data were collected by staff working on behalf of a law enforcement agency as part of the academy process. The YMCA step test was completed in an indoor basketball stadium the week prior to academy as part of a larger fitness assessment of the recruits [29]. Prior to the YMCA step test, each recruit’s age, height, and body mass were recorded. Height was measured using a portable stadiometer (seca, Hamburg, Germany), while body mass was recorded by electronic digital scales (Omron Healthcare, Kyoto, Japan). Recruits generally did not eat in the 2–3 hours prior to their testing session which featured the YMCA step test as they were completing employee-specific documentation for the agency. The circuit training sessions were performed outdoors in the first two weeks of an 8-week academy during a physical training session between 0600 and 0800. The circuit training sessions were solely the responsibility of the law enforcement agency training staff. The researchers had no input into the design or implementation of these sessions. The weather conditions for the circuit training sessions were typical of the southern California climate [17,32].

### 2.3. YMCA Step Test

The YMCA step test was administered as a fitness assessment to measure aerobic capacity, and the data was also used to determine the CA recruit fitness groups. Administration of the YMCA step test followed standard procedures [33,34,35,36]. This test has been used to assess aerobic fitness in other tactical populations, including active duty military personnel [21] and firefighters [37], highlighting its applicability for CA recruits. Further, the YMCA step test has face validity for those working in custody facilities as climbing stairs is a daily job task for CAs. The test was performed on a basketball court, with approximately 12 inch (~31 cm) high bleacher seats used for the step. Although it could have been beneficial to customize step heights to all recruits, this was not realistic within the training and testing environment for CA recruits, as a result of the logistics surround law enforcement training academies [18]. Recruits completed the step test in groups of 6–8, such that they could be paired up with a tester to measure their recovery HR.

To complete the YMCA step test, CA recruits stepped in time to a 96 beats per minute metronome continuously for 3 minutes. The beat was played from an iPad handheld device (Apple Inc., Cupertino, California) connected to a portable speaker (ION Block Rocker, Cumberland, Rhode Island) positioned on a higher bleacher seat in front of the recruits. Following the 3 minutes, recruits immediately sat on the step while recovery HR was manually taken by a staff member via the carotid or radial artery for 60 seconds [35,36].

### 2.4. Circuit Training

The circuit training sessions were designed by the academy training staff, and were a mix of calisthenics and running exercises. The exercises the recruits completed in small groups are shown in Table 1. Work periods were approximately 60 seconds in length, with between-exercise transition/recovery times of approximately 30 seconds. The total time for each session was approximately 60–70 minutes. Exact times for each circuit cannot be detailed, as the staff adapted to and responded to recruit behavior. This was done because of the nature of the training sessions, where exercise techniques were taught or reinforced. Maximal effort was expected from each exercise, and this was reinforced from stern instructions provided by training staff. This was in part done to encourage maximum effort, but also stress inoculation, which is typical within law enforcement training academies [30,38]. Nonetheless, the two circuit training sessions were representative of the approach adopted within physical training for this law enforcement agencies’ academy training.

### 2.5. Heart Rate Measurement

HR data were collected with a HR monitor (Zephyr Performance Systems, Annapolis, MD, USA) that was fitted into a chest strap worn under the physical training attire for each recruit. Previous research has shown that this system provides a valid and reliable measure of HR during exercise [39,40]. The HR-monitoring system featured in this study has also been used in other tactical populations including air force operators [41] and firefighters [42], which highlights its application to CA recruits. Training staff fitted the recruits with the chest strap and HR monitor prior to the two circuit training sessions. As described by Harry and Booysen [43], HR (measured in beats per minute) was captured at a frequency of 250 Hz via electrode sensors that detected *r* waves of the QRS complex.

After the circuit training sessions, HR data were downloaded and exported to Microsoft Excel (Microsoft Corporation Redmond, Washington, USA) as a comma-separated value file at a sampling rate of 1 second [43]. Start and finish times for the training sessions were noted, and used to remove the data not related to the circuit training sessions [43]. Data were analyzed relative to exercise intensity zones defined according to the American College of Sports Medicine (ACSM) [44]. The training zones were: very light (<57% of maximum HR [HRmax]), light (57–63% HRmax), moderate (64–76% HRmax), vigorous (77–95% of HRmax) and very vigorous (>95% HRmax) [44]. Similar to Johnson et al. [42], HRmax was calculated using the age-predicted maximum HR formula: *220*—*the CA recruit’s age*. The percentage of HRmax was calculated using the formula: *HR* (*beats per minute*) ÷ *HRmax* × *100* [43]. Peak HR, mean HR, and mean percentage of HRmax achieved in each circuit training session, in addition to the total time and percentage of total time spent in each of the training zones for the two sessions were, recorded for statistical analysis.

### 2.6. Statistical Analysis

Statistics were computed using the Statistics Package for Social Sciences (Version 26.0; IBM Corporation, New York, USA). Descriptive statistics (mean ± standard deviation (SD)) were used to profile all variables. The CA recruits were divided into three groups based upon YMCA step test recovery HR using a percentile split [45]. The top 25% (lowest recovery HR following the YMCA step test) were placed in the HF group; the bottom 25% (highest recovery HR following the YMCA step test) were placed in the LF group; and the remaining recruits were allocated to the MF group. This split was implemented to ensure clear differences between the HF and LF groups [45], such that a more definitive analysis of the potential influence of aerobic fitness on HR response to the circuit training sessions could be conducted. A one-way analysis of variance, with Bonferroni post hoc for multiple comparisons, was used to calculate any significant (*p* < 0.05) differences between the groups in peak HR, mean HR, mean percentage of HRmax, and time spent and percentage of total time in the different HR zones during each circuit training session. The two sessions were analyzed separately. The sexes were combined within the groups as all recruits completed the same circuit regardless of sex. Furthermore, combining the data from the two sexes was appropriate as there are no separate graduating standards for male and female CA recruits from this agency or across the state. Previous law enforcement research has also combined the sexes in data analysis [8,28,45,46,47,48,49].

## 3. Results

Table 2 details the descriptive data for age, height, and body mass for the different groups, in addition to the recovery HR from the YMCA step test. There were no significant differences between the groups in age (*p* < 0.535) or body mass (*p* < 0.530). The MF group was significantly taller than the LF group (*p* = 0.033). With regards to the YMCA step test, the HF group had a recovery HR significantly lower than the MF and LF groups; the MF group’s recovery HR was lower than the LF group (*p* < 0.001). 

**Figure 1:**
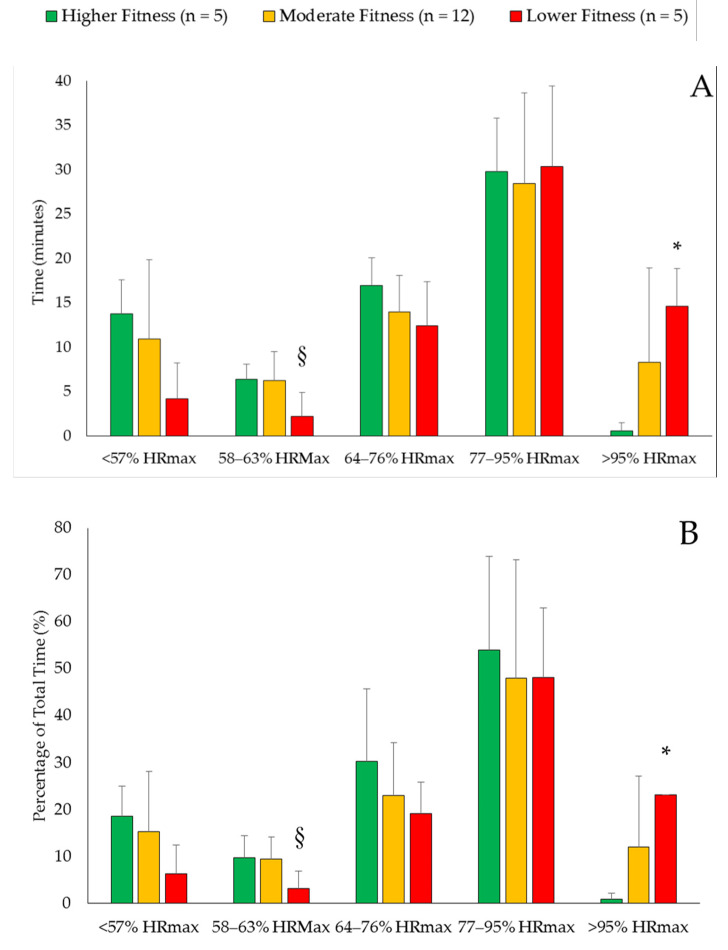
Time spent (**A**) and percentage of total time spent (**B**) in heart rate (HR) intensity zones defined by the American College of Sports Medicine [44] for higher fitness, moderate fitness, and lower fitness custody assistant recruits during circuit training session one. * Significantly (*p* < 0.05) different from the HF group. § Significantly (*p* < 0.05) different from the MF group.

**Figure 2:**
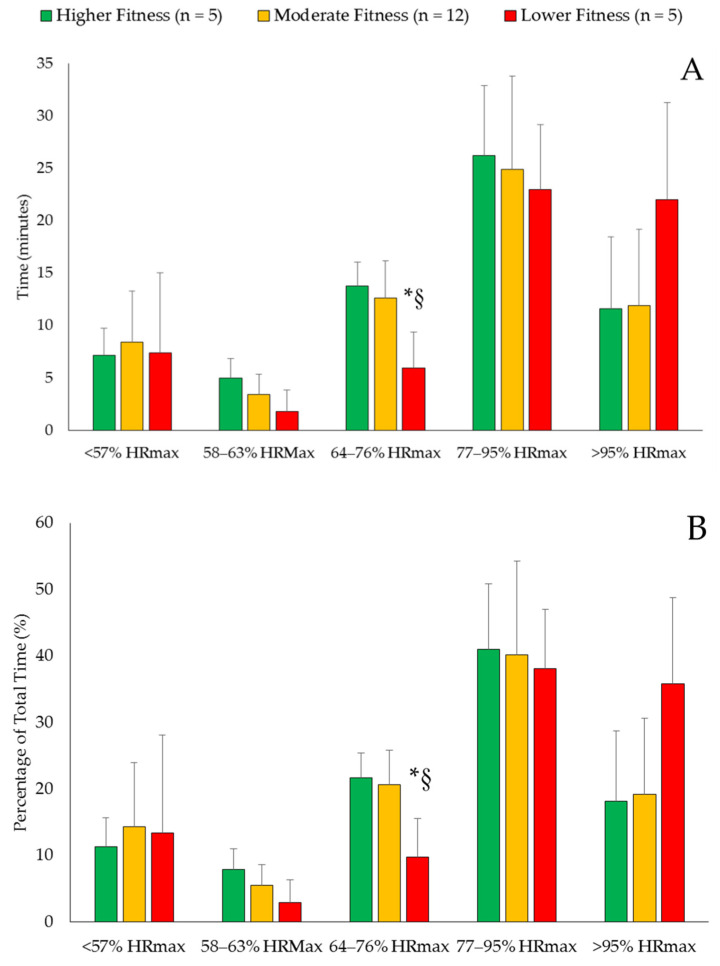
Time spent (**A**) and percentage of total time spent (**B**) in heart rate (HR) intensity zones defined by the American College of Sports Medicine [44] for higher fitness, moderate fitness, and lower fitness custody assistant recruits during circuit training session two. * Significantly (*p* < 0.05) different from the HF group. § Significantly (*p* < 0.05) different from the MF group.

Table 3 displays the peak HR, mean HR, and mean percentage of HRmax for the three groups in the two circuit training sessions. There were no significant between-group differences in peak HR, mean HR, and mean percentage of HRmax in either of the circuit training sessions (*p* = 0.120–0.974). Figure 1 displays the time and percentage of total time spent in the different HR intensity zones for each group in the first circuit training session. Figure 2 displays the same information for the second circuit training session. In session one, the MF group spent a significantly longer duration in the light training zone for time (*p* = 0.047) and percentage of time (*p* = 0.028) compared to the LF group. The LF spent a significantly longer time (*p* = 0.047) and percentage of time (*p* = 0.027) in the very vigorous zone compared to the HF group. In session two, the LF group spent significantly more time in the moderate training zone compared to both the HF and MF groups (*p* = 0.002–0.004). The LF also spent a greater percentage of total time in the very vigorous training zone compared to the MF group (*p* = 0.044).

## 4. Discussion

This study investigated the effects of aerobic fitness on the HR responses of CA recruits to two circuit training sessions. The recruits defined as HF, MF, and LF according to their recovery HR from the YMCA step test. The YMCA step test was used as a measure of aerobic fitness because of the wide variability in fitness in CA recruits [3], with some demonstrating poor aerobic fitness [29]. A maximal aerobic fitness test, such as the multistage fitness test [7,14,17,45,50,51,52,53,54,55], may have increased the injury risk to some CA recruits because of its requirements to turn sharply [54]. As a result of the costs associated with losing a recruit because of injury [7], staff preferred the YMCA step test. Nevertheless, this test provides a valid measure of aerobic fitness [33,34], and has been adopted in other tactical professionals [21,33,34,35,36,37]. The results indicated that there were relatively few differences in HR responses and the time spent in different HR training zones between the different recruit groups. This may have been a function of the sample size for this study (HF and LF groups only had five subjects each). However, the differences that did exists in the two sessions tended to indicate that the LF recruits were experiencing greater training stress than the HF and MF recruits. Previous research has recommended the use of ability-based physical training during academy for CA recruits [1,3]; these data provide support to these suggestions.

Circuit training is commonly used within tactical populations [19,20,21], often because of the logistics and challenges present in academy physical training [18]. If circuit training is correctly implemented, this modality can lead to improvements in strength and aerobic fitness [24,25]. This study focused on potential aerobic benefits for CA recruits. The ACSM suggests that 30–60 minutes a day of purposeful moderate exercise (64–76% HRmax), or 20–60 minutes a day of vigorous exercise (77–95% of HRmax), is sufficient to elicit aerobic adaptations in adults [44]. In session one, all recruits spent enough time above the vigorous exercise threshold (HF = ~30 minutes; MF = ~34 minutes; LF = ~45 minutes) to potentially elicit aerobic fitness improvements. Additionally, the mean percentage of HRmax fell within the moderate exercise training zone for all groups. This was also the case for session two (time spent in vigorous exercise training zone: HF = ~38 minutes; MF = ~37 minutes; LF = ~45 minutes). The mean percentage of HRmax also fell within the moderate exercise training zone for all groups, although it was less than circuit training session one. Nonetheless, the data would suggest that if circuit training sessions such as these two were implemented consistently over the course of a CA training academy, it should lead to aerobic capacity improvements in many recruits. The practical application of this finding is that circuit training sessions can be designed such that they will elicit physiological responses that could lead to improvements in aerobic fitness if consistently applied during academy training.

However, what was noticeable in the data analyzed in this study was the longer duration of time spent by the LF recruits in the vigorous-to-very vigorous training zones compared to the HF and MF recruits. This would suggest that the two circuit training sessions were more challenging for these recruits. Thus, it should be discussed whether these training intensities were appropriate for the LF recruit group. In session one, the LF recruits spent significantly less time in the light training zone compared to the MF recruits, and more time in the very vigorous training zone compared to the HF recruits. In session two, the LF recruits spent more time in the moderate training zone compared to both the HF and MF recruit groups, and a greater percentage of total training time in the vigorous zone compared to the MF recruits. Across the two sessions, these data showed that the LF recruits appeared to be working harder in both circuit training sessions compared to HF and MF recruits. This supports previous findings in tactical research. For example, in Australian Army recruits during a group endurance marching session, Orr and Moorby [56] found some recruits had a HR above 180 beats per minute, while others were closer to 150 beats per minute. Viewed in broader context, the cumulative effect of continually working harder for every physical training session warrants consideration, as does the potential downstream impacts of this workload on the LF recruits when completing other tasks (e.g., defensive tactics) during the day. If the LF recruits are fatigued from circuit training sessions earlier in the day, they may perform poorer in scenario-based training because of incomplete recovery. This could be an issue, as recruits can be separated from a law enforcement training academy if they fail aspects of their scenario-based training [14]. A practical application of this data is that HR measurements could provide an indication of whether some CA recruits are experiencing greater challenges during physical training in a way that could affect their ability to graduate (i.e., overtraining, increased risk of injury) [7,57]. 

Notably, this study detailed variation in the exercise intensity experienced by recruits. This is indicative of “one-size-fits-all” training models, where less fit individuals will find the training more difficult than fitter individuals. Although staff may use a paramilitary model to incorporate socialization aspects to training [30,38], this could limit some of the fitness adaptations experienced by recruits. Those recruits with higher fitness may find a circuit training session less challenging, while those recruits with lesser fitness may find sessions too difficult. Making ability-based modifications to training in law enforcement recruits can reduce the risk of overtraining and injury, which can occur when recruits work beyond their physical capacity [7,57]. Granted, training staff can encounter many challenges when attempting to implement individuated training, both from a logistical and staff perspective [18]. However, there are some modifications training staff could make to their circuit training to utilized a more ability-based focus. For example, Moreno et al. [19] detailed a circuit-based training session where ability-based modifications can be made to exercises, such that all recruits can complete the “same” exercise with differences in intensity (e.g., partner-assisted vs. normal vs. partner-resisted push-ups; partner-assisted vs. normal vs. weighted pull-ups). The data suggested law enforcement agency training staff should explore these options if they utilize circuit training for their recruits. The appropriate programming and periodization of circuit training (and other training modalities) during academy can lead to significant improvements in a range of physical fitness qualities for law enforcement personnel, including muscular endurance, strength, and power, and anaerobic and aerobic capacity [58,59]. The results from this study provide further evidence for the need for law enforcement staff to explore ability-based exercise modifications for recruits in their academy training classes.

What was also interesting in the data from this study was the peak HR achieved by the recruits from each group. Across both sessions, the recruits were close to or above 180 beats per minute, which would imply that there were periods of maximum effort completed by the recruits. Additionally, these peak HR responses in the circuit training sessions were similar to those recorded during a defensive tactics scenario for CA recruits [6]. In a sample of 19 CA recruits, Lockie et al. [6] found that 13 recruits had a peak HR of 170 beats per minute or above. In firefighters, Abel et al. [20] analyzed circuit training see whether this training method could elicit similar physiological responses to strenuous job tasks. Abel et al. [20] noted the importance of investigating this concept, as it was important to ensure any training practices adopted actually prepared firefighters for their typical job tasks. The data in this study suggested that circuit training could be designed to elicit similar heart rate responses to physically demanding tasks for CAs, in this instance the defensive tactics scenario detailed by Lockie et al. [6]. This is notable, given that defensive tactics and inmate restraint is one of the key occupational tasks for CAs and correctional staff [1,2,3,4,5,6]. Future research should detail whether the HR and physiological responses to academy training (including circuit training) matches the demands of custody job tasks to best optimize the physical preparedness of recruits. 

There are study limitations that should be detailed. The sample size was relatively small, and the HF and LF groups only featured five recruits each. Nonetheless, as a result of the dearth of research that focuses on the fitness of CAs and correctional populations (despite their importance to law enforcement agencies) [1,2,3,4,5,6,10], it is essential that research such as the current investigation are conducted and released. Although the results could be assumed (i.e., less fit recruits find physical training harder), this type of research is essential to lay the platform for evidence-based practice in law enforcement physical training. This is especially important given that, as noted earlier, culture and tradition may drive training approaches in law enforcement academies [18,30]. Future research should investigate the HR responses for a larger sample of CA recruits to further build this evidence base. The training staff did not use a maximal exercise test, such as the multistage fitness test, to measure aerobic fitness in the CA recruits [7,14,17,45,50,51,52,53,54,55]. However, the reasoning behind this were presented earlier in the discussion. Further to this, the YMCA step test does provide a valid measure of aerobic fitness [33,34], and thus was appropriate for this study. Recovery HR measured from the YMCA step tests was measured manually by multiple testers [35,36]. It would have been beneficial to have access to and use the HR monitors to measure recovery HR, which has been done in other studies [21,37]. However, they were only available for the circuit training sessions. There could have been some variation in the actual HR versus measured HR of the CA recruits. Nonetheless, the recovery HR data from the YMCA step test did result in clear delineations between the fitness groups, which would suggest that HR was measured accurately. Due to the nature of law enforcement training academies, it is likely that the HR responses of the CA recruits was not just influenced by the circuit training exercises, but also the stress imposed by training staff [30,38]. Nonetheless, the data provided by the agency was an accurate representation of the training experiences of CA recruits, and thus provides practical application.

## 5. Conclusions

CA recruits with lower aerobic fitness measured by YMCA step test recovery HR tended to be working at a higher intensity in the analyzed circuit training sessions compared to recruits with superior aerobic fitness. Although all recruits spent enough time in the analyzed sessions above the vigorous training threshold where they could experience aerobic fitness improvements [44], the LF recruits did appear to find the training more difficult. Over the long term, this may be problematic for these recruits as they may be working beyond their physical capacity, and could increase their risk of overtraining and injury [7,57]. In support of previous recommendations [1,3,18], the data from this study suggest that training staff explore the use of ability-based training modifications within circuit training for CA recruits during academy.

## Figures and Tables

**Table 1:** List of exercises from the two circuit training sessions.

Circuit Training Session One	Circuit Training Session Two
50 m jog	Push-ups
Submaximal effort sprints	805 m (880-yard) run
Body weight side lunges	Shadow boxing
Body weight walking lunges	Crunches
Bear crawl	402 m (440-yard) run
Duck walks	Mountain climbers
Side stepping/squat shuffling	201 m (220-yard) run

**Table 2:** Descriptive data (mean ± SD) for age, height, body mass, and recovery heart rate (HR) from the YMCA step test for higher fitness (HF), moderate fitness (MF), and lower fitness (LF) custody assistant recruits.

Variables	HF (n = 5)	MF (n = 12)	LF (n = 5)
Age (years)	26.20 ± 3.03	28.92 ± 7.35	25.80 ± 7.76
Height (m)	1.66 ± 0.03	1.71 ± 0.06	1.62 ± 0.05 §
Body Mass (kg)	69.90 ± 12.10	67.67 ± 11.46	65.49 ± 11.22
YMCA Step Test Recovery HR (beats)	113.60 ± 4.39	134.00 ± 7.31 *	156.00 ± 5.52 *§

* Significantly (*p* < 0.05) different from the HF group; § Significantly (*p* < 0.05) different from the MF group.

**Table 3:** Descriptive data (mean ± SD) for peak heart rate (HR), mean HR, and mean percentage of age-predicted heart rate maximum (%HR Max) for higher fitness (HF), moderate fitness (MF), and lower fitness (LF) custody assistant recruits in the two circuit training sessions.

Variables	HF (n = 5)	MF (n = 12)	LF (n = 5)
**Circuit Training Session One**			
Peak HR (beats per minute)	179.60 ± 7.89	197.00 ± 23.78	207.60 ± 20.19
Mean HR (beats per minute)	132.84 ± 5.98	138.93 ± 15.37	148.52 ± 8.60
Mean percentage of HRMax (%)	74.00 ± 2.69	71.19 ± 9.70	72.07 ± 7.95
**Circuit Training Session Two**			
Peak HR (beats per minute)	213.20 ± 23.78	210.58 ± 21.84	211.60 ± 18.26
Mean HR (beats per minute)	145.47 ± 11.73	137.34 ± 20.08	147.63 ± 25.31
Mean percentage of HRMax (%)	68.91 ± 9.10	65.81 ± 11.17	70.00 ± 12.61
